# A head-to-head comparison of the EQ-5D-3L index scores derived from the two EQ-5D-3L value sets for China

**DOI:** 10.1186/s12955-022-01988-w

**Published:** 2022-05-19

**Authors:** Ruo-Yu Zhang, Wei Wang, Hui-Jun Zhou, Jian-Wei Xuan, Nan Luo, Pei Wang

**Affiliations:** 1 Shanghai Centennial Scientific Co., Ltd, Shanghai, China; 2 School of Public Health, Fudan University, 130 Dong An Road, Shanghai, 200032 China; 3 Business School, University of Shanghai for Science and Technology, Shanghai, China; 4 Health Economic Research Institute, Sun Yat-Sen University, Guangzhou, China; 5 Saw Swee Hock School of Public Health, National University of Singapore, Singapore, Singapore; 6 Key Lab of Health Technology Assessment, National Health Commission of the People’s Republic of China (Fudan University), Shanghai, China

**Keywords:** EQ-5D-3L, China, Value set, Index score, Comparison

## Abstract

**Objective:**

Two EQ-5D-3L (3L) value sets (developed in 2014 and 2018) co-exist in China. The study examined the level of agreement between index scores for all the 243 health states derived from them at both absolute and relative levels and compared the responsiveness of the two indices.

**Methods:**

Intraclass correlations coefficient (ICC) and Bland–Altman plot were adopted to assess the degree of agreement between the two indices at the absolute level. Health gains for 29,403 possible transitions between pairs of 3L health states were calculated to assess the agreement at the relative level. Their responsiveness for the transitions was assessed using Cohen effect size.

**Results:**

The mean (SD) value was 0.427 (0.206) and 0.649 (0.189) for the 3L_2014_ and 3L_2018_ index scores, respectively. Although the ICC value showed good agreement (i.e., 0.896), 88.9% (216/243) of the points were beyond the minimum important difference limit according to the Bland–Altman plot. The mean health gains for the 29,403 health transitions was 0.234 (3L_2014_ index score) and 0.216 (3L_2018_ index score). The two indices predicted consistent transitions in 23,720 (80.7%) of 29,403 pairs. For the consistent pairs, Cohen effective size value was 1.05 (3L_2014_ index score) or 1.06 (3L_2018_ index score); and the 3L_2014_ index score only yielded 0.007 more utility gains. However, the results based on the two measures varied substantially according to the direction and magnitude of health change.

**Conclusion:**

The 3L_2014_ and 3L_2018_ index scores are not interchangeable. The choice between them is likely to influence QALYs estimations.

## Introduction

The EQ-5D-3L (3L) is one of the most widely used utility instruments in measuring health-related quality of life (HRQoL) [1–4] for use in quality-adjusted life years (QALYs) calculation. It has a classification system consisting of five dimensions: mobility (MO), self-care (SC), usual activities (UA), pain/discomfort (PD), anxiety/depression (AD), with three functioning levels (no problems, some problems, and extreme problems) in each dimension. The system thus defined 243 (3^5^) possible health states [5], and each of them can be coded into a five-digit number ranging from “11111” to “33333” (e.g., 12321 means no problems in mobility, some problems in self-care, extreme problems in usual activities, some problems in pain/discomfort and no problems in anxiety/depression). A single utility index score can be assigned to each health state by using a value set, which was developed in a valuation study based on general population’s health preferences. Since health preferences differ across populations [6, 7], a number of 3L value sets have been derived in different countries/regions [8]. Some countries (e.g., Korea, USA, and China) even developed two value sets due to respective reasons [9–14]. Taking China for example, compared to the first value set developed in 2014 (i.e., 3L_2014_ value set) using a sample comprising residents mainly form urban areas, the second value set developed in 2018 (i.e., 3L_2018_ value set) adopted a more representative sample of residents from both rural and urban areas (Table 1).

**Table 1:** Comparison of valuation method and characteristics of the two EQ-5D-3L value sets for China

	3L_2014_	3L_2018_
*Valuation method*		
Sample size used	1222 respondents	6000 respondents
Sampling area	Beijing, Shenyang, Nanjing, Chengdu, and Guangzhou (Urban area)	Jiangsu, Guangdong, Hebei, Chongqing, and Shaanxi (One rural and one urban area)
Time of data collection	2011.03.11–05.25	2014.07.10–08.25
Sampling method	Quota sampling	A multistage, stratified, clustered random sampling
Number of health states directly valuated	97	43
Number of health states valued by each respondents	13	13
Valuation protocol used	Paris protocol	MVH protocol
Modeling approach	Ordinary least squares; weighted least squares	Ordinary least squares; general least squares; weighted least squares
Choice of final model	An ordinary least square model including 10 dummies with constant and N3	An ordinary least square model including 10 dummies without constant and N3
*Characteristics of the two value sets*		
The range of index scores	[−0.149, 1]	[0.170, 1]
The median of index scores	0.427	0.653
Number of health states worse than dead (%)	6 (2.5%)	0 (0%)
Dimension importance order	MO, PD, SC, AD, UA	SC, MO, AD, UA, PD
Scoring parameter	1−(0.039 + 0.099*MO2 + 0.246*MO3 + 0.105*SC2 + 0.208*SC3 + 0.074*UA2 + 0.193*UA3 + 0.092*PD2 + 0.236*PD3 + 0.086*AD2 + 0.205*AD3 + 0.022*N3)	1−(0.077*MO2 + 0.267*MO3 + 0.044*SC2 + 0.291*SC3 + 0.037*UA2 + 0.054*UA3 + 0.027*PD2 + 0.041*PD3 + 0.036*AD2 + 0.177*AD3)

Paris protocol: a successor of the MVH protocol for valuation of EQ-5D-3L health states

*MVH* The Measurement and Valuation of Health protocol

*TTO* time trade-off

*MO* mobility; SC: self-care; UA: usual activities; PD: pain/discomfort; AD: anxiety/ depression; N3: if any level 3 problems were present in a state

2: level 2 problems; 3: level 3problems

For instance, the utility score for “22213” was 1–0.039–0.099–0.105–0.074–00.205–0.022 = 0.456 (3L_2014_ value set)

Despite the availability of the EQ-5D-5L (5L, a new version of 3L) index score with improved psychometric properties [15–18], the 3L index score is still with great usefulness due to the considerations of consistency and continuity in decision making process [19]. Indeed, the National Health Service Survey in China continually used the 3L to measure the HRQoL of Chinese residents even after the publication of the 5L value set for China in 2017 [20]. Moreover, the 3L can also be used to generate the 5L index score based on the 5L information and a crosswalk function [21], thus utilizing the advantages of 5L descriptive system.

Similarly, the 3L_2014_ value set is still more frequently used than the 3L_2018_ value set, albeit with its disadvantage in the sampling method. According to Web of Science, the former has been cited in 139 articles by April 16, 2021, 62 of which cited it after the availability of the latter. In contrast, the 3L_2018_ value set has been cited only eighteen times since its publication [22, 23]. Given the noticeable differences in coefficients of scoring algorithms for the two value sets (Table 1), it is unlikely that the two value sets would yield identical utility index scores for the same health state. However, it remains unclear to what extent the use of different utility scores generated from the two value sets would affect results of QALYs computation, which mainly depends on the difference in utility scores rather than the absolute utility scores. Moreover, it is not known whether the difference in the utility scores is clinically important as well. Our previous study has compared the two 3L indices in diabetes patients, and found that they had different discriminative power and the choice between them may impact the QALYs estimation [24]. Another study has also compared them in patients with gastric cancer and healthy controls, and showed that the 3L_2014_ index score had better ability to distinguish the patients from controls [25]. A study published in Chinese also compared the two 3L value sets in measuring the HRQoL of Tibet residents and concluded they could not be used interchangeably [26]. Nevertheless, all the previous studies were based on either a single disease group or a special group, it is not known whether the findings could be generalized to general populations or other patients in China.

Hence, the study aimed to: (1) examine the level of agreement at both absolute and relative levels of all the 243 index scores derived from the two 3L value sets for China; and (2) compare the responsiveness of two indices (i.e. to capture the real changes in health states over time).

## Methods

### The two 3L indices generated from the two 3L value sets for China

The two 3L value sets were developed using different sampling methods, valuation protocols [27, 28], modeling methods, leading to distinct algorithms for calculating the 3L index scores (Table 1). For example, the utility score for health state “23221” is 0.466 (i.e., 1-0.039-0.099-0.208-0.074-0.092-0.022) according to the 2014 algorithm or 0.568 (i.e., 1-0.077-0.291-0.037-0.027) according to the 2018 algorithm. In the study, both algorithms were used to generate the two index scores of all the 243 3L health states for analysis. There are three main differences between them. First, for the 3L_2014_ value set, respondents were selected from urban areas through a quota sampling; while for the 3L_2018_ value set, a more representative sample of respondents were obtained from both rural and urban areas by using a random sampling method. Second, the 3L_2014_ and 3L_2018_ value sets were developed using the Paris protocol and the Measurement and Valuation of Health (MVH) protocol, respectively, whereby the former protocol is an improvement of the latter. Third, the time-trade off (TTO) technique for the 3L_2014_ value set was based on the ‘death’ state to elicit health utility scores, but not for the 3L_2018_ value set. Those differences led to distinct algorithms for calculating the 3L index scores (Table 1).

### Statistical analysis

We assessed the distributions of the two indices (i.e., 3L_2014_ index score and 3L_2018_ index score) using the Shapiro–Wilk test. T-test or Wilcoxon rank-sum test were then used to compare their mean values wherever appropriate.

A two-way mixed intraclass correlation coefficient (ICC) [29] and Bland–Altman plot [30] were adopted to assess the degree of agreement between the two indices at absolute level. The agreement was considered good when the ICC value was higher than 0.7. The Bland–Altman plot was used to visualize and assess the level of agreement across different utility segments, whereby the Y-axis depicts the differences in score between the two indices, and the X-axis represents their mean values. A limit of 0.074, that is the minimally important difference (MID) of the 3L index score, [31] was used to determine whether the magnitude of the difference would be clinically important.

To examine the agreement of the two 3L index scores at relative level, we simulated all the possible health states transitions that may occur over time. All the 243 health states were paired to form 29,403 (C^2^_243_) health state combinations, each of which was used to simulate a pair of health states before and after treatment. It was assumed that the health states with higher index scores were as the states after treatment (post-treatment), and the lower were as the health states before treatment (pre-treatment) [32]. Hence, the health gains of our simulated treatment were always positive. However, the index score of the same health state may vary when changing from one value set to the other, thus a health state labeled as pre-treatment when using the 3L_2014_ value set may represent post-treatment instead when using the 3L_2018_ value set in the same pair, or vice versa. This was what we considered as an “inconsistent” pair of health states [33], whereby the choice of index scores would have a substantial impact on health outcomes, i.e. one may generate a positive health gain, while the other may result in health losses.

On the contrary, for a “consistent” pair, the health state representing pre-treatment remained unchanged regardless of using either the 3L_2014_ or 3L_2018_ value set. Given the magnitude of health gains may vary from one value set to another, the consistent group was further divided into four subgroups according to the perceived direction and magnitude of the change before and after treatment: (1) major improvement (i.e. at least one dimension in the health transition is increased from level 3 to level 1 or level 2, and no dimension is decreased); (2) minor improvement (i.e. at least one dimension in the health transition is increased from level 2 to level 1, and no dimension is increased from level 3 to 1 or 2, nor is the level of any dimension decreased); (3) mixed response with minor deterioration (i.e. at least one dimension is decreased from level 1 to 2 and no dimension is decreased from level 1 or 2 to 3); (4) mixed response with major deterioration (i.e. at least one dimension is decreased from level 1 or 2 to 3) [33]. It should be noted that, if the level of one dimension deteriorates yet the level of the others improves in a health transition, it would be considered as a mixed response with some deterioration and thus assigned to either subgroup 3 or 4. We then compared the health gains yielded from the two 3L indices for all the transitions, consistent transitions, and each subgroup of the consistent transitions.

Moreover, in order to help understand how a single-level change in severity of descriptive systems would result in different utility change between the two value sets, we computed changes in utility values between pairs of adjacent health states for each value set. We called them “adjacent” when two health states are exactly the same except for one dimension where the severity level differs by one only [15, 34–36]. For example, health states “21111” and “11111” were considered adjacent.

We also compared the responsiveness of the two 3L indices within the consistent group by using Cohen effect size [37]^.^ It is commonly used to measure the effect size of a treatment, and is independent of the sample size which is unlike the significance test. It is calculated as the difference in the mean scores between post-treatment and pre-treatment divided by the standard deviation of the pre-treatment. The effect size was categorized as small (0.2–0.5), moderate (> 0.5–0.8), or large (> 0.8) [37]. Given that the hypothetical treatment was fixed in our simulation, the effect size would reflect the ability of an index score to discern changes in two known health states. The higher the effect size, the more responsive the index score is. We calculated and compared Cohen effect size for all the consistent pairs and each subgroup of the pairs. Microsoft Excel and Stata and SAS were used for statistical analysis.

## Results

**Fig. 1:**
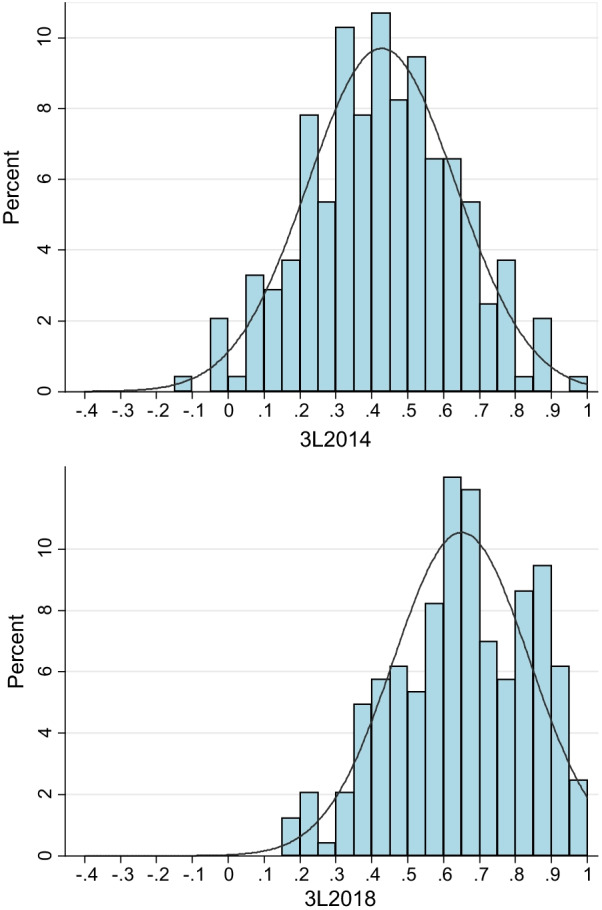
Histograms of utility values generated from the two EQ-5D-3L value sets

**Fig. 2:**
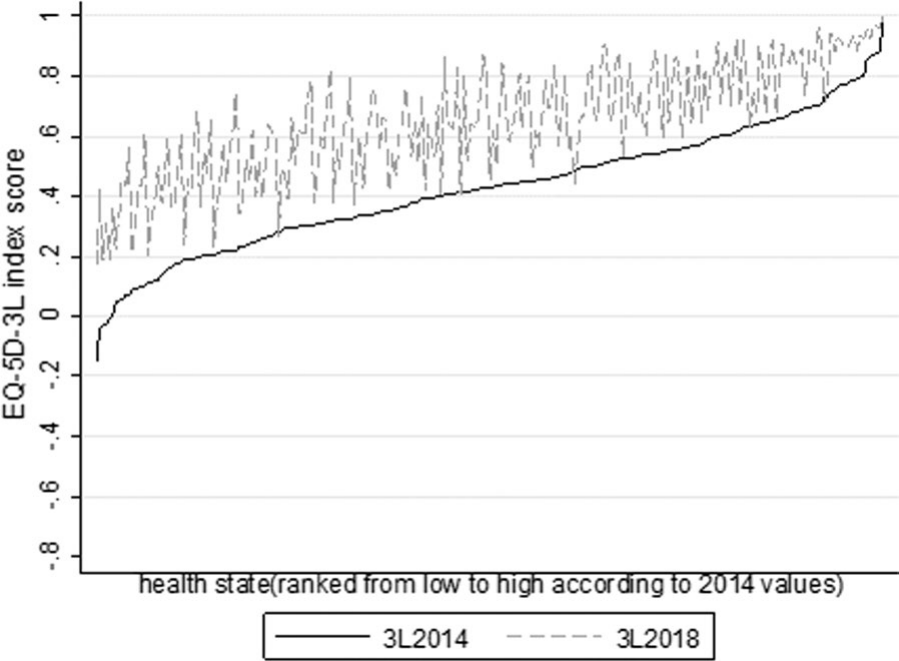
EQ-5D-3L index scores for 243 EQ-5D health states produced by the two value sets

**Fig. 3:**
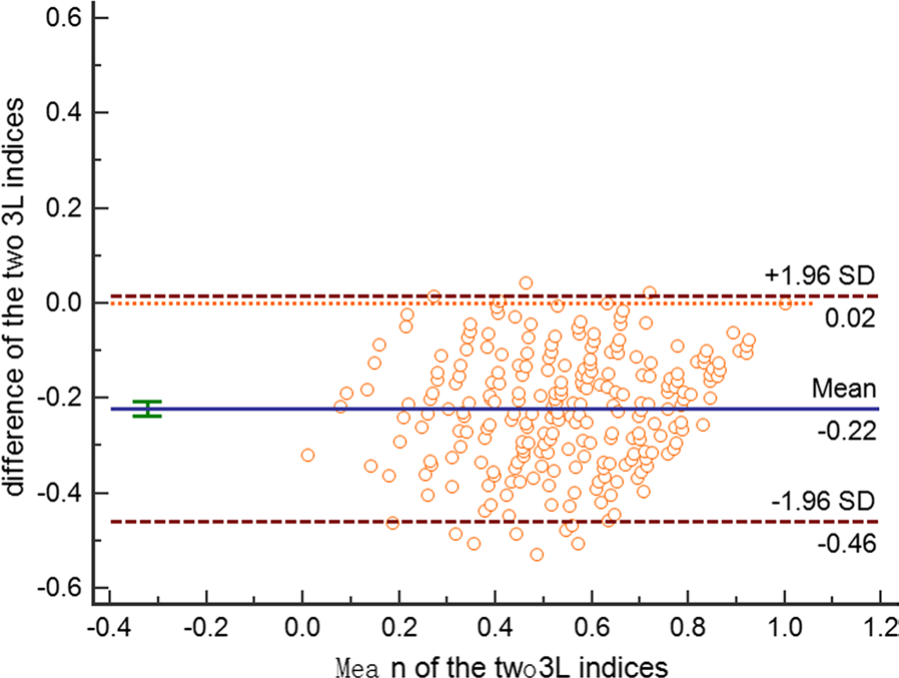
Bland–Altman Plot of EQ-5D-3L Index Scores generated by the two value sets

The two 3L indices were both normally distributed according to the Shapiro–Wilk test (Fig. 1). Overall, the 3L_2014_ value set generated systematically lower index scores compared with those yielded from the 3L_2018_ value set. The mean (SD) value of all the index scores was 0.427 (0.206) for the former and 0.649 (0.189) for the latter, with the difference in mean being 0.222 (*p* < 0.001) (Table 2); the 3L_2014_ value set also had lower scores for 239 out of 243 health states. Meanwhile, the difference and variance between the two index scores were not invariant but generally increased with the increasing in health-state severity (Fig. 2). For example, the index score of the second-best health state was 0.887 (for state “11211”) and 0.973 (for state “11121”); while the minimum index score was −0.149 and 0.170 (for the worst state “33333”) according to the 3L_2014_ or 3L_2018_ value set, respectively. Although the overall agreement between the two kinds of index scores was good (ICC = 0.896), 88.9% (216/243) of the points were beyond the MID limit according to Bland–Altman plot (Fig. 3).

**Table 2:** Comparison of the two EQ-5D-3L index scores at absolute and relative levels

	n	Mean	SD	Minimum	Maximum
*EQ-5D-3L index score*					
3L_2014_	243	0.427	0.206	−0.149	1
3L_2018_	243	0.649	0.189	0.170	1
3L_2014_–3L_2018_	243	−0.222	0.121	−0.529	0.043
*Health gains from transitions*					
3L_2014_	29,403	0.234	0.173	0	1.149
3L_2018_	29,403	0.216	0.158	0	0.830
3L_2014_-3L_2018_	23,720*	0.007	0.152	−0.521	0.529

3L:EQ-5D-3L

*number of consistent pairs

On the other hand, the difference between the two indices was not so obvious for the 29,403 health transitions: the mean differences (SD) were 0.234 (0.173) and 0.216 (0.158) for the 3L_2014_ and 3L_2018_ index scores, respectively. Similarly, in 23,720 (80.7%) of 29,403 transitions, the two indices generated consistent results for health gains before and after a simulated treatment, with the difference in mean health gains for the transitions being only 0.007 (*p* < 0.001) (Table2). Among the consistent transitions, the number of pairs for each subgroup was 6752 (major improvement), 781(minor improvement), 4515 (mixed response with minor deterioration), and 11,672 (mixed response with major deterioration).

In the subgroups of major/minor improvement, the 3L_2014_ index score yielded greater magnitude of health gains at 0.411/0.151(vs. 0.310/0.072 from the 3L_2018_ index score). However, it generated similar or lower health gains compared to the 3L_2018_ index score in the subgroups of “mixed response with minor deterioration” (health gains: 0.246 for both index scores) and “mixed response with major deterioration” (health gains: 0.069 vs. 0.118) (Table 3)_._ The absolute change in utility values between any two adjacent states computed using the 3L_2014_ value set was larger than that using the 3L_2018_ value set, expect for pairs that involve a change between level 2 and 3 in either “mobility” or “self-care” dimension (Table 4). Essentially, it reflected the fact that differences between coefficients of the same dimension in the scoring algorithm vary from one value set to another.

**Table 3:** Responsiveness of the two EQ-5D index scores in simulated transitions between EQ-5D-3L health states

	All Consistent Transitions (n = 23,720)		Major Improvement (n = 6752)		Minor Improvement (n = 781)		Mixed Response with Minor Deterioration (n = 4515)		Mixed Response with Major Deterioration (n = 11,672)	
	3L_2014_	3L_2018_	3L_2014_	3L_2018_	3L_2014_	3L_2018_	3L_2014_	3L_2018_	3L_2014_	3L_2018_
Mean (SD)Pre-treatment score	0.329 (0.193)	0.552 (0.184)	0.213 (0.173)	0.486 (0.180)	0.450 (0.172)	0.692 (0.159)	0.374 (0.166)	0.570 (0.173)	0.370 (0.186)	0.574 (0.179)
Mean (SD)Post-treatment score	0.531 (0.188)	0.748 (0.158)	0.624 (0.182)	0.796 (0.151)	0.601 (0.193)	0.765 (0.170)	0.620(0.150)	0.815 (0.123)	0.439 (0.156)	0.692 (0.153)
Mean (SD) Health gains	0.203 (0.244)	0.195 (0.215)	0.411 (0.169)	0.310 (0.168)	0.151 (0.073)	0.072 (0.039)	0.246 (0.156)	0.246 (0.152)	0.069 (0.224)	0.118 (0.230)
Cohen Effect size	1.05	1.06	2.38	1.72	0.88	0.45	1.48	1.42	0.37	0.66

**Table 4:** Differences in utility change of adjacent health states between two value sets

EQ-5D-3L state*	3L_2014_ value set		3L_2018_ value set	
	Utility value	Change†	Utility value	Change†
11,111	1.000		1.000	
21,111	0.862	0.138	0.923	0.077
31,111	0.693	0.169	0.733	0.19
11,111	1.000		1.000	
12,111	0.856	0.144	0.956	0.044
13,111	0.731	0.125	0.709	0.247
11,111	1.000		1.000	
11,211	0.887	0.113	0.963	0.037
11,311	0.746	0.141	0.946	0.017
11,111	1.000		1.000	
11,121	0.869	0.131	0.973	0.027
11,131	0.703	0.166	0.959	0.014
11,111	1.000		1.000	
11,112	0.875	0.125	0.964	0.036
11,113	0.734	0.141	0.823	0.141

*For illustration, only some adjacent health states are presented to reflect that a single “one-level” change in the 3L descriptive system would result in a change in utility values

†Column “change” lists all the possible absolute changes in utility values between any pair of adjacent health states for each value set

The two indices also showed a similar level of sensitivity to change for all the consistent changes, with Cohen effect size values at 1.05 and 1.06, respectively. Nevertheless, the value varied substantially across the subgroups. In the subgroups of major/minor improvement, the 3L_2014_ index score demonstrated higher values than the 3L_2018_ index score (Cohen effect size: 2.38 vs. 1.72/0.88 vs. 0.45). While in the subgroup of mixed response with major deterioration, the result was reversed (Cohen effect size:0.37 vs. 0.66); in the subgroup of mixed response with minor deterioration, the two index scores demonstrated similar responsiveness with Cohen effect sizes at 1.48 vs. 1.42. (Table 3).

## Discussion

In the study, we compared the agreement of all the two 3L index scores generated from the two 3L value sets for China. We found that the 3L_2014_ index score was systematically lower than the 3L_2018_ index score at absolute level, but their differences at relative level varied in terms of the direction and magnitude of the health change.

It is not surprising that the 3L_2014_ index score was much lower given the 3L_2014_ algorithm has larger values in 8 out of 10 parameters and two more terms (i.e., constant and N3) further pulling down the scores (Table 1). The difference and variance between the two index scores were also increased with the increasing in health-state severity. Regarding the former, the difference in level-3 (L3) parameters between the two algorithms is in general larger than the difference in level-2 (L2) parameters. This, plus the use of N3 term, lead to the increased difference. The latter could be ascribed to the fact that the 3L_2018_ algorithm has two L3 parameters with larger values (i.e., MO3 and SC3) than those of the 3L_2014_ algorithm. As a result, for health states including the problems, the difference between the index scores may be reduced rather than increased, resulting in larger variance for all health states including L3 problems. Difference in algorithm parameters may be attributed to several factors such as the valuation protocol, modeling method, as well as the sample used [13, 14]. The sample for the 3L_2018_ algorithm including the rural population, who may be more likely to live with economic hardships over years. Hence, they may be able to endure more pain and suffering, leading to a relatively higher estimate in utility values for health problems than the better-off residents. In addition, the 3L_2018_ value set used an open-ended TTO question. The developers of the 3L_2018_ value set believed that due to cultural reasons, death is a taboo in China, especially in rural areas. When using the TTO method, the interviewers did not tell the respondents to imagine die immediately after living in a hypothetical health state for a period of time. Therefore, the respondents may make variant assumptions about the length of life and health states of the continued lives, which may have led to an overestimation of the TTO.

The two indices generated consistent results for the majority (80.7%) of health transitions. For the transitions involving improvement only, the results would always be consistent regardless the differences in scoring algorithms. On the other hand, the inconsistent results would be presented for the transitions including both improvement and deterioration in different dimensions. Compared to the 3L_2014_ algorithm, the parameter coefficients of the 3L_2018_ algorithm display greater variance. Its parameter value for L2 and L3 problems of the 3L_2018_ algorithm varied from 0.027 (PD2) to 0.077 (MO2), and 0.041 (PD3) to 0.291(SC3); while such the parameters for the 3L_2014_ algorithm ranged from 0.074 (UA2) to 0.099 (MO2) and 0.205(AD3) to 0.246 (MO3). For example, a health transition resulted from health state “11131”to “11113” would be considered as health gain and health loss according to the 3L_2014_ (0.031) algorithm and 3L_2018_ (−0.136) algorithm, respectively.

With regard to all the consistent health transitions, both the index scores showed similar health gains and responsiveness, but they varied considerably across the four subgroups. The health gains and responsiveness of the 3L_2014_ index score were found to be better or greater than those of the 3L_2018_ index score in the “major improvement” and “minor improvement” subgroups, which suggested that the use of the 3L_2014_ algorithm would tend to result in larger QALY gains for the two subgroups. On the other hand, in the subgroups of “mixed response with minor deterioration” and “mixed response with major deterioration”, the two index scores generated similar or even reversed results. For the subgroups 1 & 2, the 3L_2014_ algorithm overall has larger parameter values, indicating the health gain from a transition from extreme/some problems to no problems is much greater according to it. Similarly, the magnitude of difference between L2 and L3 parameters is also generally larger for the 3L_2014_ algorithm, leading to comparable conclusions for the transitions from extreme problems to some problems. This point became clearer when we compared changes in utility values of two adjacent health states between the two value sets, as shown in Table 4. For the subgroups 3 & 4, the 3L_2014_ algorithm has relatively similar parameter values across the five L2 and the five L3 parameters. Hence, for a health transition involving both improvement and deterioration, the magnitude of health gain from the improvement in a certain dimension may be offset to a large extent by the deterioration from another dimension according to the 3L_2014_ algorithm. The resulting health gains and responsiveness were therefore not larger or better than those based on the 3L_2018_ algorithm in the subgroups.

It should be bear in mind that in reality the frequencies of the 243 health states and 29,403 transitions would be distributed disproportionately. For example, the state “11111” has been the most frequently observed in a number of studies in China, which may lead to different conclusions. [24] When measuring individuals who are expected to be either stable or gain improvement in all the 5 dimensions of 3L from an intervention, the 3L_2014_ value set may be a more preferable choice. But in other scenarios, the choice becomes less straightforward and thus it is recommended to apply both value sets in data analyses as part of a robustness check. Also, the absolute utility score could also influence the QALY calculation to some extent. Hence, more empirical studies are warranted to further assess the impact in various settings in China.We also acknowledge a new 3L value set for China’s rural population developed by Liu et al. has been available recently [38]. They also found that the utilty scores generated from the value set were generally lower than those of the two 3L value sets used in the current analysis. We did not include the value set as we have finished the analysis and paper writing before its publication. Nevetherless, the differences among the three kinds of 3L utilities may necessitate the valuation of 5L health states from both rural and urban respondents since the current 5L value set for China is based on urban respondents only.

## Conclusion

Our results suggested a substantial difference between the 3L_2014_ and 3L_2018_ index scores at absolute level; while their differences at relative level differed according to the type of health change. Our findings suggested that choosing which value set to generate 3L index score is very likely to influence QALYs estimate in China.

## Data Availability

The data that support the findings of this study are available on request from the corresponding author.

## Abbreviations

3L: EQ-5D-3L
5L: EQ-5D-5L
AD: Anxiety/depression
HRQoL: Health-related quality of life
ICC: Intraclass correlations coefficient
MID: Minimally important difference
MO: Mobility
MVH: Measurement and valuation of health protocol
PD: Pain/discomfort
QALYs: Quality-adjusted life years
SC: Self-care
TTO: Time-trade off
UA: Usual activities
